# Epidemiological study of metabolic syndrome in Brazilian soldiers

**DOI:** 10.20945/2359-3997000000115

**Published:** 2019-03-18

**Authors:** Marcos de Sá Rego Fortes, Samir Ezequiel da Rosa, Walmir Coutinho, Eduardo Borba Neves

**Affiliations:** 1 Instituto de Pesquisa da Capacitação Física do Exército Rio de Janeiro RJ Brasil Instituto de Pesquisa da Capacitação Física do Exército (IPCFEx), Rio de Janeiro, RJ, Brasil; 2 Pontifícia Universidade Católica do Rio de Janeiro Pontifícia Universidade Católica do Rio de Janeiro Rio de Janeiro RJ Brasil Pontifícia Universidade Católica do Rio de Janeiro (PUC-Rio), Rio de Janeiro, RJ, Brasil

**Keywords:** Metabolic syndrome, abdominal obesity, dyslipidemia, hypertension, hyperglycemia

## Abstract

**Objective:**

The aim of this study was to carry out an epidemiological analysis of metabolic syndrome among Brazilian Army soldiers.

**Subjects and methods:**

Two thousand seven hundred and nineteen male soldiers of the Brazilian Army were evaluated from 2014 to 2016. Characteristics: age = 27.77 (± 8.59) years and BMI = 25.15 (± 3.41) kg/m^2^. Blood tests and anthropometric measures were performed following the criteria of the International Diabetes Federation Task Force on MS Epidemiology and Prevention, 2009. The epidemiological analysis was based on Odds ratio (OR) with confidence interval (CI).

**Results:**

The prevalence of MS found was 12.21%. Both WC and BMI proved to be good predictors of changes in MS physiological markers. Increased WC and BMI were strongly associated with all physiological markers. Soldiers with WC ≥ 90 were more likely to present MS with OR = 33.37 (24.37-45.7). Soldiers with WC ≥ 90 also presented high risk of: high triglycerides with OR = 5.98 (4.69-7.61); low HLD-c with OR = 1.78 (1.47-2.16); and increased systolic blood pressure OR = 3.10 (2.55-3.76). Soldiers with BMI ≥ 30 had a high risk of: increased glucose with OR = 2.69 (1.93-3.75); and increased diastolic blood pressure with OR = 3.02 (2.22-4.10).

**Conclusion:**

Both WC and BMI can be considered as good predictors of changes in MS physiological markers. We believe that WC and BMI should be used as screening tools to indicate the soldiers that must undergo blood tests to monitor MS prevalence.

## INTRODUCTION

The increase of the global burden of Non-Communicable Chronic Diseases (NCDs) is a public health problem worldwide. NCDs are multifactorial in nature and share several modifiable risk factors such as smoking, physical inactivity, inadequate diet, obesity, dyslipidemia and alcohol consumption (1–3).

The increasing incidence of overweight and obesity represents a serious public health problem with implications for society and health systems (4). It contributes to the morbidity and mortality by Diabetes Mellitus, Heart Disease, Stroke and Cancer (5). It has been suggested that overweight and obesity, in addition to causing these diseases, can also significantly reduce life expectancy.

An study developed by Chaves and cols. (6), including the years 1980-2005, and using the Brazilian Army database with 8.989.508 teenagers aged 17-19 years, showed an increasing trend of overweight and obesity prevalence in all Brazilian states, In that period, the number of teenagers with overweight increased three-fold and with obesity, six-fold. Another study (7) with Brazilian militaries showed the following percentages of obese or overweight subjects: 12.91%, 7.98% and 17.84%, considering the Body mass index (BMI), waist/ hip ratio (WHR) and waist circumference (WC), respectively. The best anthropometric indicator for systemic arterial hypertension was the waist/hip index (Odds Ratio = 4.45, 1.45-13.17).

Metabolic syndrome (MS) is a complex disorder, characterized by a cluster of cardiovascular risk factors, such as high blood pressure, central fat deposition, dyslipidemia and insulin resistance (8–10), MS includes a constellation of cardiovascular and type 2 diabetes risk factors. Its etiology involves complex interactions between genetics, metabolism and environmental factors, including diet. Its prevalence has increased in developed and developing countries, in males and females, and in adults, teenagers and children (11). Vidigal and cols. (12) identified, in a recent systematic review, MS prevalence in the Brazilian adult population varying between 28.9% and 29.6%, according to the criterion used to define it. Half the studies have used the NCEP-ATP III (2001) criterion for MS diagnosis.

Although MS has appeared in many forms and definitions for more than eight decades, only in the last five years, the real controversy over its definition and meaning emerged (13). It is defined in many ways by: NCEP/ATP III (National Cholesterol Education Program Expert Panel on Detection, Evaluation, and Treatment of High Blood Cholesterol in Adults); IDF (International Diabetes Federation) and WHO (World Health Organization) among others (14).

Different organs adopt different criteria to define MS and the cut-off points for its components. In 2009, a new definition of MS was presented as a consensus between IDF and AHA/NHLBI after a meeting between their respective representatives aiming at arriving at a common denominator for its definition, since there was divergence between the two criteria. In this manner, the main characteristic of this new criterion is that obesity, defined by the cut-off point of the abdominal circumference, by ethnic groups, is no longer a prerequisite. Obesity, however, remains an important risk factor to be investigated (9).

Surveys conducted by IPCFEx (Brazilian Army Research Institute of Physical Fitness) show that from the 17^th^ BRABAT to the 21^st^ BRABAT, the percentage of soldiers with MS increased from 3.2% to 10.9%. Another study involving 1,383 men (18 to 62 years old) allotted in military organizations of Grande Natal – RN, conducted with soldiers from the Brazilian Navy found prevalence of 17.6% using IDF criteria (15). The anthropometric indices (body mass index, waist circumference and waist-hip ratio) are the most widely used methods for measuring general and abdominal obesity in large epidemiological studies. Body mass index is used to measure general adiposity, while waist circumference and waist/hip ratio measure abdominal adiposity. There is a correlation between BMI, WC and the total mass of the adipose tissue.

Haghighatdoost and cols. (16) point out that general obesity measured by the BMI is a known risk factor for diabetes. Although BMI is frequently claimed as a simple measure to determine disease risk, it has several constraints: it does not differentiate lean mass from fat mass nor fat distribution. It is important to mention that visceral adiposity plays a more important role in the development of insulin resistance and diabetes, than general adiposity (17). Therefore, WC was adopted as a measure of abdominal adiposity that takes into consideration fat distribution. In this sense, WC has been considered better than BMI in estimating intra-abdominal adipose tissue and provides a measure of body fat distribution (18–20). In this sense, the aim of this study was to carry out an epidemiological analysis of metabolic syndrome among Brazilian Army soldiers, considering these anthropometrics index as risk factors.

## SUBJECTS AND METHODS

A cross-sectional observational study was conducted adopting a quantitative approach of MS prevalence and its components, according to the criteria of the Joint Scientific Statement (9). It evaluated 2,719 male soldiers from the Brazilian Army, serving in all regions of the country, from 2014 to 2016. The present study was registered at the National System of Ethics in Research (SISNEP, Portuguese acronym), submitted and approved by the Ethics Committee of the Marcílio Dias Naval Hospital, under no. 1.551.242, CAAE no. 47835615.5.0000.5256. Data collection was divided into two days. On the first day, an anthropometric evaluation was carried out, determining body mass (BM) with the soldier in the orthostatic position, barefoot, using only a swimsuit, with the aid of a Lider digital analytical scale, P150M model, with 200 kg maximum load and increments of 0.1%.

The height was measured with a metallic stadiometer, brand Sanny. The soldiers stood erect, with the arms extended and attached to the trunk, feet joined and keeping contact with the stadiometer by the heel, with the head oriented in the Frankfurt plane. The measures were performed in respiratory apnea, aiming at minimizing possible instabilities of the vertebral column.

The WC was measured with a 2M anthropometric tape from Sanny medical, Starret, model SN 4010. The soldiers remained standing, with relaxed abdomen, arms extended and weight equally distributed between the legs, with the feet near and parallel. The measurement was taken at the end of the expiration, taking care not to compress the skin, at the midpoint between the last rib and the iliac crest. The anthropometric variables were obtained following recommendations of Lohman and cols. (21).

On the second day, the biochemical tests of MS markers (TG, HDL-C, GLUC) were performed. Samples were collected in IPCFEx clinical analysis laboratory, strictly following the collection recommendations of the Brazilian Society of Clinical Pathology/Laboratory Medicine from 2010 and the resolution of the Collegiate Board – RDC no. 306/2004 – ANVISA for sample managing and disposal. A hemodynamic evaluation (SAP, DAP) was conducted next as prescribed in VI Guidelines for the use of Ambulatory Arterial Pressure Monitoring published by the Brazilian Society of Cardiology 2010. The parameters of the Joint Scientific Statement (9) were used for MS diagnosis. All participants were informed of the procedures and risks to which they would be submitted and signed a Free Informed Consent Term.

### Statistics

A descriptive and exploratory analysis was carried out to characterize the population under study according to their demographic characteristics. Univariate analysis was performed to examine the associations of the variables investigated, odds ratios (OR) and respective confidence intervals (CI) were calculated. The receiver-operating characteristic (ROC) curve analysis was used to determine cut-off points of an index composed by the sum of excess in BMI and WC, and their sensitivity and specificity values to identify MS subjects.

## RESULTS

The following table presents the characteristics of the variables related to the sample’ MS, expressed as mean and standard deviation (Table 1).

**Table 1:** Anthropometric characteristics and risk factors for MS of male soldiers of the Brazilian Army, serving in all regions of the country, from 2014 to 2016. (n = 2,719)

	Age (years)	WC (cm)	BMI	SAP (mmHg)	DAP (mmHg)	GLUC (mg/dL)	TG (mg/dL)	HDL-c (mg/dL)
Mean	27.8	88.9	25.1	120.3	77.2	88.9	92.0	47.0
S.D.	8.6	13.9	3.4	10.1	8.8	13.9	57.4	13.1

Values expressed in absolute numbers. S.D.: standard deviation.

All values obtained are within the normality range (Table 2).

**Table 2:** MS prevalence in Brazilian soldiers, with central obesity (WC) and BMI who participated in the UN peacemaking mission in Haiti between 2014 and 2016. (n = 2,719)

	Absolute Frequency	Relative Frequency
Effective soldiers	2,719	100,00%
MS	332	12.21%
N MS	2,387	87.79%

The table above shows low prevalence when compared to that of the Brazilian civilian population, which, according to national studies ranges between 28.9 and 29.6% (Table 3).

**Table 3:** Groups of soldiers with and without metabolic syndrome and WC (n = 2,719)

WC	With MS	Without MS	Total
Altered WC	278	319	597
Not altered WC	54	2,068	2,122
Total	332	2,387	2,719

Odds ratio: 33.37 (CI = 24.37 – 45.7).

Estimated OR was 33.37, i.e., the probability of presenting the syndrome was 34 times higher in exposed individuals (altered WC) than in those that were not exposed (not altered WC) (Table 4).

**Table 4:** Group of soldiers with and without metabolic syndrome and BMI (n = 2,719)

WC	With MS	Without MS	Total
Altered BMI	122	91	213
Not altered BMI	210	2,296	2,506
Total	332	2,387	2,719

Odds ratio: 14.66 (confidence interval from 10.79 to 19.91).

Estimated OR was 14.66, i.e., the probability to develop the disease was approximately 15 times higher in exposed individuals (altered BMI) than in those that were not exposed (not altered BMI) (Table 5).

**Table 5:** Prevalence (P), odds ratio (OR) and confidence interval of MS risk factors in the group of soldiers (n = 2,719)

	Altered	n	P (%)	OR (IC 95%)
Altered WC	SAP	257	9.5	3.10 (2.55 – 3.76)
	DAP	153	5.6	2.39 (1.91 – 3.00)
	GLUC	129	4.7	2.47 (1.94 – 3.14)
	TG	183	6.7	5.98 (4.69 – 7.61)
	HDL	220	8.1	1.78 (1.47 – 2.16)
Altered BMI	SAP	99	3.6	2.84 (2.15 – 3.77)
	DAP	70	2.6	2.96 (2.18 – 4.03)
	GLUC	55	2.0	9.62 (7.17 – 12.91)
	TG	69	2.5	2.26 (1.51 – 2.76)
	HDL	82	3.0	1.74 (1.31 – 2.32)

The table above shows that increased WC and BMI were strongly associated with all physiological markers. Soldiers with increased WC presented high risk of increase of altered SAP with OR = 3.10 (2.55 – 3.76); altered DAP with OR = 2.39 (1.91 – 3.00); altered GLUC with OR = 2.47 (1.94 – 3.14); altered TG with OR = 5.98 (4.69 – 7.61); altered HDL-c with OR = 1.78 (1.47 – 2.16).

Soldiers with altered BMI presented high risk of increase of altered SAP with OR = 2.84 (2.15 – 3.77); altered DAP with OR = 2.96 (2.18 – 4.03); altered GLUC with OR = 9.62 (7.17 – 12.91); altered TG with OR = 2.26 (1.51 – 2.76); altered HDL-c with OR = 1.74 (1.31 – 2.32).

The comparison of BMI with WC, shows that GLUC and TG presented the greatest differences. Individuals with altered WC have 2.5 times more probability of presenting altered GLUC, while those with altered BMI have approximately 10 times more chances of presenting altered GLUC. Concerning TRIG, with altered WC, the probability is approximately 6 times, while with altered BMI the probability is 2.3 times.

It was found good values of sensibility and specificity to MS when analyzed BMI and WC together, through a simple index that was calculated by the sum of excess in each variable, in relation their cut-off points. The values of sensibility and specificity for each cut-off point, of the cited index, are presented in the Table 6.

**Table 6:** Values of sensibility and specificity to MS when analyzed BMI and WC together soldiers (n = 2,719)

Sum of excess in BMI and WC, in relation their cut-off points	Sensibility	Specificity
-38.01	1.00	0.00
-14.49	0.99	0.30
-12.39	0.98	0.37
-10.13	0.97	0.46
-8.47	0.96	0.53
-6.88	0.95	0.59
-0.71	0.90	0.80
0.02	0.89	0.82
0.37	0.88	0.83
0.59	0.87	0.83
1.21	0.86	0.84
1.48	0.85	0.85
2.48	0.80	0.87
3.21	0.75	0.88
4.26	0.70	0.90
5.19	0.65	0.91
6.01	0.60	0.92
6.97	0.55	0.93
8.09	0.50	0.94
9.40	0.45	0.95
10.57	0.40	0.96
11.72	0.35	0.97
13.91	0.30	0.98
15.62	0.25	0.99
17.87	0.20	0.99
22.26	0.13	1.00

## DISCUSSION

MS is defined as a constellation of interconnected physiopathological, biochemical, clinical and metabolic factors that directly increase the risk of cardiovascular disease, type 2 diabetes mellitus and all-cause mortality. Insulin resistance, visceral adiposity, atherogenic dyslipidemia and high blood pressure are some of the various factors that constitute the syndrome (22–24). According to Stern and cols. (25), the risk of diabetes is increased 5-fold in people with MS.A possible explanation is lipotoxicity predisposing to beta cell dysfunction, in addition to insulin resistance, core pathophysiological element of metabolic syndrome (26).

The prevalence of metabolic syndrome found in the present study was 12.21%. According to Bortoletto and cols. (24), a systematic review among the adult population worldwide showed high prevalence of MS, ranging from 25% to 35%, with higher frequency in women. A cross-sectional study conducted by Costa and cols. (15), involving about 1.400 individuals from the Brazilian Navy and using the criterion of the International Diabetes Federation (IDF) for MS diagnosis, as in the present research, found prevalence of 17.6%. All specific combinations of risk factors for MS that exceeded the expected prevalence presented abdominal obesity as one of its components. Those authors also reported that central obesity appeared as the most common factor in combinations with prevalence higher than expected, which seems to corroborate its importance in MS phenotype.

The prevalence of hypertension, type 2 diabetes mellitus, dyslipidemia and MS is greater in individuals with high values of WC, than in those with normal values within the same category of BMI (27). In the present study, 22% of the sample presented altered WC that is above the cut-off point. Of those, 10.2% were classified as syndromic. In addition, it was found that the OR of those subjects with altered WC developing MS was approximately 34 times higher than the OR of individuals with WC below the cut-off point. On the other hand, the OR of individuals with altered BMI was 15 times.

Among the measures of obesity, WC and BMI were significantly associated with all risk factors for MS. BMI was more significant for GLUC than WC, while the latter correlated mainly with TG. Indeed, among the various categories of BMI, those within the category of normal WC presented substantially higher amounts of abdominal fat, which consists, almost entirely of visceral fat, than those within the category of low WC.

Regarding the hemodynamic parameters, in the present study, the soldiers with increased WC presented high risk of altered SAP increase with OR = 3.10 and altered DAP with OR = 2.39.

Levine and cols. (28) examined the prevalence of hypertension (systolic AP ≥ 140 mmHg, or diastolic AP ≥ 90 mmHg, or use of self-reported antihypertensive drugs) by BMI and WC, focusing on moderate WC, in a large national biracial cohort of American adults aged between 45 and 84 years. They found prevalence of hypertension of 44% of those with low WC, 55% (women < 80 cm; men < 94 cm), with moderate WC (women 80-88 cm; men 94–102 cm) and 66% with high WC (women > 88 cm; men > 102 cm).Moderate WC was associated with prevalence of arterial hypertension regardless the BMI in middle-aged adults and the elderly. The difference was that in the present study the blood pressure was measured separately, i.e., systolic and diastolic.

Janssen and cols. (27) grouped 14.924 adult participants by BMI and WC in accordance with the cut-off points of the “National Institutes of Health”. The OR for hypertension, diabetes, dyslipidemia and MS were calculated. With some exceptions, among the three BMI categories, those with high WC values were increasingly more predisposed to have hypertension, diabetes, dyslipidemia and MS than those with normal WC values. These results are in agreement with those found in the present research.

Regarding the association between WC and GLUC, the present study found OR of 2.47 for individuals with altered WC. Gill and cols. (29) investigated the independent contributions of WC, physical activity, and sedentary behavior to GLUC in South Asians living in Scotland. In the multivariate regression analysis, WC was significantly associated with fasting GLUC concentration, result that corroborates the findings of the present study.

Another study conducted by Mainous and cols. (30) concluded that among individuals within a healthy BMI range, the prevalence of pre-diabetes substantially increases with abdominal obesity. Similar result was found by Haghighatdoost and cols. (16).

On the other hand, Yang and cols. (31) aimed to find the best anthropometric indicator to discriminate the dyslipidemia prevalent in a non-obese Chinese population aged over 40 years. The BMI showed a higher correlation with lipids than WC and waist/hip ratio, not only in the stepwise regression analysis, but also in logistic regression. The study suggested that the BMI also played an important role in the classification of risk of adverse lipid concentration in non-obese individuals. In addition, BMI was more associated with low HDL-C e less associated with total cholesterol, regardless of gender, age and prevailing MS. In the present study, individuals with altered BMI and with altered TG and HDL-c had 3-fold greater probability of developing MS than those with normal concentration of those lipids.

Some authors have used the receiver-operating characteristic (ROC) curve analysis to determine optimal cut-off points for BMI and WC in relation to the area under the curve (AUC), sensitivity and specificity to metabolic syndrome screening. However, few of them studied these two variables combined (32–34). Takahashi and cols. (34) studied 3,915 men and 2,032 women and conclude that when combined BMI and WC, the sensitivity for the MS diagnosis increased to more than 80%. This results are in agreement with those presented in the present study that describe, i.e., a cut-off point (1,48 as Sum of excess in BMI and WC, in relation their cut-off points) which shows 85% of sensibility and 85% of specificity.

We concluded that BMI and WC are good predictors of MS and that the additional risk to health explained by WC probably reflects its capacity to act as a substitute for abdominal fat, particularly, visceral fat. Our findings suggest that BMI and WC should be used as screening tools to indicate blood test to monitor MS prevalence.

## References

[1] Bicalho PG, Hallal PC, Gazzinelli A, Knuth AG, Velásquez-Meléndez G. Atividade física e fatores associados em adultos de área rural em Minas Gerais, Brasil. Rev Saúde Pública. 2010;44(5):884-93. 10.1590/s0034-89102010005000023 20676590

[2] Malta DC, Moura ECd, Castro AMd, Cruz DKA, Morais Neto OLd, Monteiro CA. Padrão de atividade física em adultos brasileiros: resultados de um inquérito por entrevistas telefônicas, 2006. Epidemiol Serv Saúde. 2009;18(1):7-16.

[3] Kyröläinen H, Santtila M, Nindl BC, Vasankari T. Physical fitness profiles of young men. Sports Med. 2010;40(11):907-20. 10.2165/11536570-000000000-00000 20942508

[4] Bahia L, Araújo DV. Impacto econômico da obesidade no Brasil. Revista HUPE. 2014;13(1):13-7.

[5] Collaboration PS. Body-mass index and cause-specific mortality in 900 000 adults: collaborative analyses of 57 prospective studies. Lancet. 2009;373(9669):1083-96. 10.1016/S0140-6736(09)60318-4 PMC2662372 19299006

[6] Chaves VLdV, Freese E, Lapa TM, Cesse EAP, Vasconcelos ALRd. Evolução espaço-temporal do sobrepeso e da obesidade em adolescentes masculinos brasileiros, 1980 a 2005. Cad Saúde Pública. 2010;26(7):1303-13. 10.1590/s0102-311x2010000700009 20694356

[7] Neves EB. Prevalence of overweight and obesity among members of the Brazilian army: association with arterial hypertension. Cienc Saúde Coletiva. 2008;13(5):1661-8. 10.1590/s1413-81232008000500029 18813666

[8] Lopes HF. Hipertensão arterial e síndrome metabólica: além da associação. Rev Soc Cardiol Estado de Säo Paulo. 2003;13(1):64-77.

[9] Alberti K, Eckel RH, Grundy SM, Zimmet PZ, Cleeman JI, Donato KA, et al. Harmonizing the metabolic syndrome: a joint interim statement of the international diabetes federation task force on epidemiology and prevention; national heart, lung, and blood institute; American heart association; world heart federation; international atherosclerosis society; and international association for the study of obesity. Circulation. 2009;120(16):1640-5. 10.1161/CIRCULATIONAHA.109.192644 19805654

[10] Alberti KGMM, Zimmet P, Shaw J. Metabolic syndrome – a new world‐wide definition. A consensus statement from the international diabetes federation. Diabet Med. 2006;23(5):469-80. 10.1111/j.1464-5491.2006.01858.x 16681555

[11] Curi R, Calder PC. Metabolic syndrome: epidemiology, pathophysiology, and nutrition intervention. J Nutr Metab. 2012;2012:584541. 10.1155/2012/584541 PMC3384958 22778922

[12] de Carvalho Vidigal F, Bressan J, Babio N, Salas-Salvadó J. Prevalence of metabolic syndrome in Brazilian adults: a systematic review. BMC Public Health. 2013;13(1):1198. 10.1186/1471-2458-13-1198 PMC3878341 24350922

[13] Eckel RH, Alberti K, Grundy SM, Zimmet PZ. The metabolic syndrome. Lancet. 2010;375(9710):181-3. 10.1016/S0140-6736(09)61794-3 20109902

[14] Penalva DQF. Síndrome metabólica: diagnóstico e tratamento. Rev Med. 2008;87(4):245-50.

[15] Costa Fd, Montenegro VB, Lopes TJA, Costa EC. Combinação de fatores de risco relacionados à síndrome metabólica em militares da Marinha do Brasil. Arq Bras Cardiol. 2011;97(6):485-92. 10.1590/s0066-782x2011005000113 22030564

[16] Haghighatdoost F, Amini M, Feizi A, Iraj B. Are body mass index and waist circumference significant predictors of diabetes and prediabetes risk: Results from a population based cohort study. World J Diabetes. 2017;8(7):365. 10.4239/wjd.v8.i7.365 PMC5507834 28751960

[17] Consortium I. Long-term risk of incident type 2 diabetes and measures of overall and regional obesity: the EPIC-InterAct case-cohort study. PLoS Med. 2012;9(6):e1001230. 10.1371/journal.pmed.1001230 PMC3367997 22679397

[18] Klein S, Allison DB, Heymsfield SB, Kelley DE, Leibel RL, Nonas C, et al. Waist circumference and cardiometabolic risk: a consensus statement from shaping America's health: Association for Weight Management and Obesity Prevention; NAASO, the Obesity Society; the American Society for Nutrition; and the American Diabetes Association. Obesity. 2007;15(5):1061-7. 10.1038/oby.2007.632 17495180

[19] Hu F. Obesity epidemiology: Oxford University Press; 2008.

[20] Neves EB, da Rosa SE, Fortes MdSR. Prevalence and anthropometric predictors of metabolic syndrome in the Brazilian military. J Sci Med Sport. 2017;20(Supplement 2):S158.

[21] Lohman TG, Roche AF, Martorell R. Anthropometric standardization reference manual: Human kinetics books Champaign; 1988.

[22] Kaur J. A comprehensive review on metabolic syndrome. Cardiol Res Pract. 2014;2014:943162. 10.1155/2014/943162 PMC3966331 24711954

[23] Rubbo-Blanco ML, Oliveira BNd, Siqueira Filho AGd, Luiz RR, Lima RdSL. Síndrome metabólica é o principal preditor de isquemia miocárdica na SPECT. Int J Cardiovasc Sci. 2015;28(3):189-99.

[24] Bortoletto MSS, de Souza RKT, Cabrera MAS, González AD. Síndrome metabólica em estudos com adultos brasileiros: uma revisão sistemática. Espaço Saúde. 2014;15(4):86-98.

[25] Stern MP, Williams K, González-Villalpando C, Hunt KJ, Haffner SM. Does the metabolic syndrome improve identification of individuals at risk of type 2 diabetes and/or cardiovascular disease? Diabetes Care. 2004;27(11):2676-81. 10.2337/diacare.27.11.2676 15505004

[26] Cusi K. The role of adipose tissue and lipotoxicity in the pathogenesis of type 2 diabetes. Curr Diab Rep. 2010;10(4):306-15. 10.1007/s11892-010-0122-6 20556549

[27] Janssen I, Katzmarzyk PT, Ross R. Body mass index, waist circumference, and health risk: evidence in support of current National Institutes of Health guidelines. Arch Intern Med. 2002;162(18):2074-9. 10.1001/archinte.162.18.2074 12374515

[28] Levine DA, Calhoun DA, Prineas RJ, Cushman M, Howard VJ, Howard G. Moderate waist circumference and hypertension prevalence: the REGARDS study. Am J Hypertens. 2011;24(4):482-8. 10.1038/ajh.2010.258 PMC3717383 21233800

[29] Gill JM, Bhopal R, Douglas A, Wallia S, Bhopal R, Sheikh A, et al. Sitting time and waist circumference are associated with glycemia in UK South Asians: data from 1,228 adults screened for the PODOSA trial. Diabetes Care. 2011;34(5):1214-8. 10.2337/dc10-2313 PMC3114490 21464463

[30] Mainous AG, Tanner RJ, Jo A, Anton SD. Prevalence of prediabetes and abdominal obesity among healthy-weight adults: 18-year trend. Ann Fam Med. 2016;14(4):304-10. 10.1370/afm.1946 PMC4940459 27401417

[31] Yang Z, Ding X, Liu J, Duan P, Si L, Wan B, et al. Associations between anthropometric parameters and lipid profiles in Chinese individuals with age≥ 40 years and BMI< 28kg/m2. PloS One. 2017;12(6):e0178343. 10.1371/journal.pone.0178343 PMC5478121 28632766

[32] Cheong KC, Ghazali SM, Hock LK, Subenthiran S, Huey TC, Kuay LK, et al. The discriminative ability of waist circumference, body mass index and waist-to-hip ratio in identifying metabolic syndrome: Variations by age, sex and race. Diabetes Metab Syndr. 2015;9(2):74-8. 10.1016/j.dsx.2015.02.006 25819369

[33] Yang J, Qiu H, Li H, Zhang Y, Tao X, Fan Y. Body Mass Index, Waist Circumference and Cut-Off Points for Metabolic Syndrome in Urban Residents in Ningxia. Open J Endocr Metab Dis. 2015;5(12):163-70.

[34] Takahashi M, Shimomura K, Proks P, Craig TJ, Negishi M, Akuzawa M, et al. A proposal of combined evaluation of waist circumference and BMI for the diagnosis of metabolic syndrome. Endocr J. 2009;56(9):1079-82. 10.1507/endocrj.k09e-197 19734693

